# Encapsulation of *Phaseolus lunatus* Protein Hydrolysate with Angiotensin-Converting Enzyme Inhibitory Activity

**DOI:** 10.5402/2013/341974

**Published:** 2013-09-24

**Authors:** Jorge Carlos Ruiz Ruiz, Maira Rubí Segura Campos, David Abram Betancur Ancona, Luis Antonio Chel Guerrero

**Affiliations:** Facultad de Ingeniería Química, Universidad Autónoma de Yucatán, Periférico Norte. Km. 33.5, Tablaje Catastral 13615, Col. Chuburná de Hidalgo Inn, CP 97203, Mérida, YUC, Mexico

## Abstract

The objective of recent research has been to seek alternative therapeutic treatments; for this reason, the use of protein hydrolysates from diverse sources has been studied. A way to guarantee that these treatments reach the site of action is through protection with covers, such as microcapsules. Therefore, proteins from the legume *Phaseolus lunatus* were hydrolyzed and encapsulated with a blend of *Delonix regia* carboxymethylated gum/sodium alginate (50 : 50 w/w). Hydrolysis release conditions in a simulated gastrointestinal system were obtained using CaCl_2_ concentrations as the main factor, indicating that lower CaCl_2_ concentrations lead to an increased hydrolysis release. Beads obtained with 1.0 mM of CaCl_2_ exhibited a better hydrolysate release rate under intestinal simulated conditions and the proteins maintained an IC_50_ of 2.9 mg/mL. Capsules obtained with the blend of *Delonix regia* carboxymethylated gum/sodium alginate would be used for the controlled delivery of hydrolysates with potential use as nutraceutical or therapeutic agents.

## 1. Introduction

Enzymatic hydrolysis of food proteins has produced various biologically active peptides with immunostimulating, opioid, antithrombotic, anticariogenic, and bactericidal or angiotensin-converting enzyme (ACE) inhibitory functions and has been the focus of recent research [1]. The renin angiotensin system plays an important role in blood pressure and in cardiac and vascular functions. Renin produces decapeptide angiotensin I from angiotensinogen. ACE catalyzes the formation of angiotensin II by cleaving the dipeptide from the C-terminal of angiotensin I in the vascular wall [2]. ACE inhibitors have been prescribed for hypertensive patients worldwide, and many clinical application data have demonstrated that ACE inhibitors significantly reduce the morbidity and mortality of patients with myocardial infarction or heart failure [3]. Thus, ACE inhibitors may induce skin rashes, angioneurotic edema, diarrhea, cough, and dizziness [4]. Because hypertensive patients often need life-long medical treatment, interest has been focused on the isolation and identification of ACE-inhibitors which may be obtained from new and varied sources like foods [5].

Legumes are widely consumed in south-eastern Mexico as a major dietary protein source in both human and animal diets. *Phaseolus lunatus* L. is distributed throughout Latin America, the southern United States, Canada, and many other regions worldwide. It is a reasonably drought-tolerant legume with reported yields as high as 1500 kg/ha and is known to be an efficient organic fertilizer. The bean of *P. lunatus* is high in protein (24%) that increases up to 70% when subjected to alkaline extraction to obtain protein concentrates [6]. Extensive hydrolysis of *P. lunatus* protein concentrates with commercial and digestive enzymes could therefore produce a number of peptides with a myriad of potential applications, for example, as a natural-source of therapeutic elements in medical treatments and/or as an ingredient in functional foods.

However, bioactive peptides orally administered to reduce blood pressure are liable to be digested and lose their activities. One of the main advantages of microsphere formulation is to provide stability for labile compounds that are rapidly degraded or cleared out *in vivo*. Cells or enzymes such as proteases in the surrounding tissues would not have contact with the protein until it is released from the microsphere matrix [7]. Microspheres could be readily injected subcutaneously or into other target sites or even given orally. In fact, developments in controlled release microsphere formulations have accelerated given the developments in polymer science and synthesis technology. The polymer must not alter the pharmacological properties of the drug and should not produce adverse side effects or any degradation product which has any other activity. It should be biocompatible, nontoxic, and nonirritant [8].

There are several natural polymers available, like starch, alginate, zein, chitin, or collagen. The use of modified polysaccharides with ionic charges to encapsulate substances with biological activity has become increasingly popular in the nutraceutical food and pharmaceutical industries. Microencapsulation protects nutraceuticals, keeps them stable in storage at room temperature, and ensures the release of an appropriate dosage at a gastric or intestinal pH [9]. *Delonix regia* trees are considered an ornamental species, and gum from its seeds has not been previously used to encapsulate nutraceutical substances in food and drugs [10]. Nonetheless, *D. regia* seed gum is potentially useful for microencapsulation because it contains galactomannan-type polysaccharides that are similar to those of guar gum (*Cyamopsis tetragonolobus*) and locust bean (*Prosopis chilensis*) gum [11]. Previous studies have demonstrated its capacity to form microcapsules as models of bioactive release molecules [12]. The few branched regions present in this flamboyant seed native gum consist of *α*-D-mannose (1 → 4) linkages and *α*-D-galactose (1 → 6) branches (mannose-galactose 2 : 1 ratio). Its mannose and galactose proportions are similar to those of guar gum but differ in terms of the OH bond position in the main chain; flamboyant gum has *α*-D-mannose while guar gum has *β*-D-mannose [13].

In the present research, to retain the ACE inhibitory activity of peptides in the gastrointestinal tract, hydrolysates of *P. lunatus* beans were encapsulated in beads of carboxymethylated *D. regia* seed gum. Subsequently, the beads *in vitro* release capacity and ACE inhibitory activity of hydrolysate was evaluated.

## 2. Materials and Methods

### 2.1. Seeds and Chemicals

*Delonix regia* seed pods were collected in Yucatan, Mexico. The collection of dry seed pods was performed approximately 100 days after the flowering period, and physically undamaged seeds no longer than 1 cm in length were removed from the sample. A total of 5 kg of seeds was collected and stored in polyethylene bags at 4°C until use. *Phaseolus lunatus* seeds were obtained from the February 2012 harvest in the state of Yucatan, Mexico. All chemicals were reagent grade from JT Baker (Phillipsburg, NJ), and pepsin was purchased from Sigma (Sigma Co., St Louis, MO, USA).

### 2.2. *D. regia* Seed Gum Extraction

Flour was produced from seed endosperm following Morochi et al. [10]. Seeds were hydrated in distilled water (1 : 5 w/v) at 70°C for 10 h. They were then wet milled (Tecator Cemotec 1090 Sample Mill, Höganäs, Sweden) to crack the seeds and free the endosperm and washed three times with distilled water to eliminate the husk and germ. The endosperm was dried at 55°C for 24 h in a circulating air oven (Imperial V Lab-Line Model 3476 M, Boston, USA) and milled (Thomas-Wiley Laboratory Mill Model 4, Swedesboro, NJ, USA), before being passed through a 20 mesh (833 *μ*m). *D. regia* gum extraction was done following Azero and Andrade [14], with some modifications. Briefly, the endosperm flour was suspended in water (1 : 30 w/v, pH 7) and heated to 50°C under constant agitation for 30 min. It was then filtered sequentially through 42 (351 *μ*m) and 100 mesh (147 *μ*m) to separate the fibrous particles from the gum. The filtered sample was precipitated in 70% (w/v) ethanol, dried at 55°C for 24 h in a circulating air oven (Imperial V Lab-Line Model 3476 M, Boston, USA), and milled (Thomas-Wiley Laboratory Mill Model 4, Swedesboro, NJ, USA) to an 80 mesh (173 *μ*m) size.

### 2.3. Carboxymethylation

The *D. regia* gum was modified by carboxymethylation using sodium chloroacetate (SCA) under heterogeneous conditions following Bahamdan and Daly [15], with some modifications. A 70 g sample of gum was allowed to swell in 400 mL of 2-propanol with nitrogen, under constant agitation (Caframo RZ-1, Heidolph, Schwabach, Germany). After initial swelling, 24.8 g of NaOH solution (40%) was added over a period of 20 min, and the mixture was left to stand for 30 min at room temperature to allow further swelling. Sixty grams of SCA solution (40%) was added over 30 min, and the mixture was allowed to react for 1 h. The reaction temperature was then raised to 70°C over 1 h, and the reaction was allowed to proceed for 3 h at 70°C. The mixture was cooled to room temperature and filtered. The resulting solid was washed and soaked in 80% (v/v) methanol/water for 30 min to remove inorganic salts. The carboxymethylated gum was recovered by filtration, washed with 99.9% methanol (Fermont 06121, Monterrey, Mexico), and dried overnight at 60°C in an oven (Imperial V Lab-Line Model 3476 M, Boston, USA).

### 2.4. Protein Concentrate

Selected grains were processed in a disk mill (model 4-E Quaker, Mill Straub Co., Philadelphia, PA, USA) until flour was produced. The flour was then sifted through 4.76 and 2.38 mm screens, and the hulls were removed with a fluidizing air bed. Samples were then milled in a Mykros impact mill until passing through a 20 mesh screen (0.85 mm) followed by milling in a Cyclotec 1093 (Tecator, Sweden) mill before being passed through a 60 mesh screen (0.24 mm). A single extraction was performed with 5 kg of flour. The flour was processed using the wet fractionation method of Betancur-Ancona et al. [6]. Briefly, whole flour was suspended in distilled water at a 1 : 6 (w/v) ratio, pH was adjusted to 11 with 1 M NaOH, and the dispersion was stirred for 1 h at 400 rpm with a mechanical agitator (Caframo Rz-1, Heidolph Schwabach, Germany). This suspension was wet milled with a Kitchen-Aid food processor, and the fiber solids were separated from the starch and protein mix by straining through 80- and 150-mesh sieves, followed by washing of the residue five times with distilled water. The protein-starch suspension was allowed to sediment for 30 min at room temperature to recover the starch and protein fractions. The pH of the separated solubilized proteins was adjusted to the isoelectric point (4.5) with 1 N HCl. The suspension was then centrifuged at 1317 ×g for 12 min (Mistral 3000i, Curtin Matheson Sci.), the supernatants were discarded, and the precipitates were freeze-dried at −47°C and 13 × 10^−3^ mbar. Until use, the protein isolate was stored in plastic containers at room temperature.

### 2.5. Enzymatic Hydrolysis

Protein concentrate hydrolysis was carried out using pepsin for 10 min, substrate concentration 4%, enzyme/substrate ratio 1 : 10, pH 2 and temperature 37°C [16]. The reaction was stopped by heating to 80°C for 20 min, followed by centrifuging at 1317 ×g for 12 min (Mistral 3000i, Curtin Matheson Sci.) to remove the insoluble portion. The degree of hydrolysis (DH) was calculated by determining free amino groups with o-phthaldialdehyde [17]: DH = (H/H_tot_)100, where H_tot_ is the total number of peptide bonds per protein equivalent, and h is the number of hydrolyzed bonds. 

### 2.6. Hydrolysate Encapsulation

Carboxymethylated gum/sodium alginate beads in a ratio 50 : 50 (w/w) were produced based on Betancur-Ancona et al. [12]. A 2^2^ factorial design with 4 central points was used (Table 1). The tested factors and levels were CaCl_2_ concentration (1 and 3 mM) and pH (4 and 10). Response variable was the amount of protein encapsulated. 150 mg of each gum and 400 mg of hydrolysate were dispersed in 25 mL of deionized water. The dispersion was dropped into a CaCl_2_ solution with a peristaltic pump at a flow of 0.17 mL/s. The beads were left to harden for 30 min.

### 2.7. *In Vitro* Release Studies

Each treatment's bead *in vitro* release capacity was evaluated with an adapted version of the method of Takagi et al. [18]. Briefly, 100 mg of dry beads was added to 50 mL beakers containing 25 mL HCl solution at pH 1.2 with 0.2 M NaCl at 37°C, to simulate gastric pH. This mixture was stirred with a magnetic stirrer at 350 rpm for 2 h on a plate shaker (Variomag Multipoint Electronic Rührer HP, Daytona Beach, Florida). The beads were recovered by decanting the solution and then placed in 50 mL beakers containing 25 mL 0.25 M phosphate buffer at pH 6.8 and 37°C, to simulate intestinal pH. This mixture was stirred with a magnetic stirrer at 1.5 g for 3 h. Protein content and residual ACE activity were calculated using 1.5 mL triplicate aliquots taken after processing with each pH system (i.e., gastric and intestinal).

### 2.8. ACE Inhibitory Activity

Angiotensin I-converting enzyme inhibitory activity was analyzed following Hayakari et al. [19]. Hippuryl-L-histidyl-L-leucine (HHL) is hydrolyzed by ACE to yield hippuric acid and histidyl-leucine. This method relies on the colorimetric reaction of hippuric acid with 2,4,6-trichloro-s-triazine (TT) in a 0.5 mL incubation mixture containing 40 *μ*mol potassium phosphate buffer (pH 8.3), 300 *μ*mol sodium chloride, 40 *μ*mol 3% HHL in potassium phosphate buffer (pH 8.3), and 100 mU/ml ACE. The mixture was incubated at 37°C for 45 min and the reaction terminated by adding TT (3% v/v) in dioxane and 3 mL of 0.2 M potassium phosphate buffer (pH 8.3). After centrifuging the reaction mixture at 10,000 ×g for 10 min, enzymatic activity was determined in the supernatant by measuring absorbance at 382 nm. All runs were done in triplicate. ACE inhibitory activity was quantified by a regression analysis of ACE inhibitory activity (%) versus peptide concentration and defined as an IC_50_ value, that is, the peptide concentration (*μ*g protein/mL) required to produce 50% ACE inhibition under the described conditions.

### 2.9. Statistical Analysis

All results were analyzed using descriptive statistics with central tendency and dispersion measures. One-way ANOVAs were run to evaluate content of protein encapsulate and ACE inhibitory activity during *in vitro* release studies. An LSD multiple range test was used to determine differences between treatments. All analyses were done according to Montgomery [20] and processed with the Statgraphics Plus version 5.1 software.

## 3. Results and Discussion 

### 3.1. *Delonix regia* Seed Gum Extraction and Carboxymethylation

*D. regia* gum extraction produced 400 g of gum per 1 kg of endosperm flour. The carboxymethylation of the gum resulted in a degree of substitution (DS) of 0.43, equivalent to 14% carboxymethyl substitution. This DS was similar than those reported for gums with a similar mannose-galactose ratios (2 : 1) in their structure, as well as guar gum (*Cyamopsis tetragonolobus*) with 0.41 [19] and 0.42 for locust bean (*Prosopis chilensis*) [11].

### 3.2. Enzymatic Hydrolysis

Hydrolysis was monitored by measuring the extent of proteolytic degradation through the degree of hydrolysis (DH) according to the o-phthaldialdehyde reaction with free amino groups. The DH produced at 10 min with pepsin was 12.4%. Extensive protein hydrolysates, that is, those with a high degree of hydrolysis (>10%), are used as protein supplements or as ingredients in special medical diets [21]. Recent research has focused on the properties of food protein-derived peptides, their biological activities, and potential health benefits. Peptides extracted from partial enzymatic hydrolysates of food proteins can provide specific health benefits, for example ACE inhibitors [22]. Peptides with biological activities have generally been isolated from food proteins via hydrolysis with digestive enzymes such as pepsin, pancreatin, trypsin, or chymotrypsin [22].

### 3.3. Hydrolysate Encapsulation

**Figure 1:**
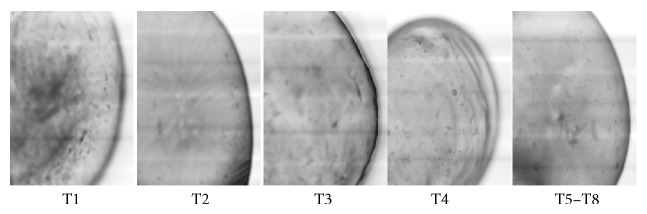
Morphology of flamboyant carboxymethylated gum/sodium alginate newly formed beads. T1 (CaCl_2_ 1.0 mM, pH 4), T2 (CaCl_2_ 3.0 mM, pH 4), T3 (CaCl_2_ 1.0 mM, pH 10), T4 (CaCl_2_ 3.0 mM, pH 10), T5–T8 (CaCl_2_ 2.0 mM, pH 7).

The reported concentrations of polymers used for gel formation vary from 1.5% to 2.5% with 0.05 to 1.5 M CaCl_2_ [23]. According to Chourasia and Jain [24], the use of polymer-blends increases the efficiency of protein delivery systems. In this study, a concentration of 3% of *D. regia* carboxymethylated gum/sodium alginate blend (50 : 50 w/w) was tested for all treatments because preliminary tests had shown that blending in this ratio improves capsule formation. The bead morphology shows that newly formed beads had a spherical shape with similar sizes (Figure 1). 

**Figure 2:**
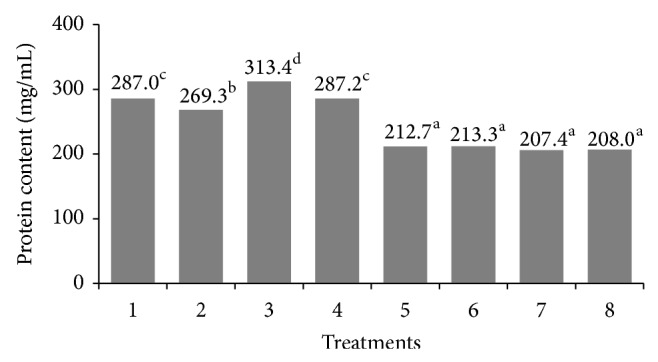
Influence of CaCl_2_ concentration and pH on the protein content in the flamboyant carboxymethylated gum/alginate beads. Different superscript letters indicate statistical difference (*P* < 0.05).

The protein content in beads ranged from 212.7 to 313.4 mg/mL (Figure 2). The higher content of protein was observed in treatment 3 and the lower content in central points. According to Figure 2, the content of protein (hydrolysate) in beads was determined by the amount of CaCl_2_ and pH with lower contents in treatments 5, 6, 7, and 8 (CaCl_2_ 2.0 mM, pH 7) and higher in treatment 3 (CaCl_2_ 1.0 mM, pH 10). According to Chandramouli et al. [25], increasing CaCl_2_ concentrations induce a more densely cross-linked gel structure and increase the content of protein encapsulated. Findings in this study were somewhat contradictory; at low concentrations of CaCl_2_ (1.0 mM), the content of protein was higher. The content of protein also was influenced by pH. At high values of pH (pH > 8), the solubility of protein was found to increase and probably showed a better interaction with the polymer blend [26]. The efficiency of protein encapsulation in beads obtained at pH 10 with 1.0 mM of CaCl_2_ was over 78%, while that in beads obtained at pH 7 with 2.0 mM of CaCl_2_ was over 53% (shown in Table 1).

### 3.4. *In Vitro* Release Studies

During the *in vitro* simulation of gastric conditions, the release of protein (hydrolysate) was detected for all bead formulations (Table 2). At pH 1.2, protein releases ranged from 10.5% to 73.1%. Treatment 2 (CaCl_2_ 3.0 mM, pH 4) released only 10.5% of the protein in 2 h, indicating the stability of beads. On the contrary, treatment 3 (CaCl_2_ 1.0 mM, pH 10) released 73% of the protein during the same time. Treatments obtained with CaCl_2_ 3.0 mM were more stable under simulated gastric conditions than those obtained with CaCl_2_ 1.0 and 2.0 mM. Using *in vitro* intestinal conditions, beads obtained with CaCl_2_ 1.0 mM at pH 4 released a higher content of protein (Table 2), which suggested that lower CaCl_2_ concentrations were needed to further enhance the extended release properties. This behavior is desirable because release in the intestinal environment (60.1%), which is where absorption takes place bioactive components, occurs with the lowest concentration of calcium, with appropriate release value in the gastric environment (16.4%).

### 3.5. ACE Inhibitory Activity

The protein concentration required to produce 50% inhibition of ACE (IC_50_) was used as an activity indicator. This indicator is expressed as mg protein/mL, with smaller values indicating a greater ACE-inhibiting power. The nonhydrolyzed *Phaseolus lunatus* protein concentrate showed no inhibitory activity on ACE. ACE inhibitory activity was generated from the *Phaseolus lunatus* protein after enzymatic hydrolysis. Hydrolysis is necessary in order to release ACE peptides from an inactive form within the *Phaseolus lunatus* protein. On the other hand, *P. lunatus* hydrolysate has good solubility in water, allowing incorporation into food matrices to generate physiologically functional foods for preventing hypertension as well as for therapeutic purposes. Treatment 3 released the highest content of hydrolysate during the gastric simulation with consequently higher ACE inhibitory activity (Table 2). Protein- and peptide-based therapeutic agents have unique physiochemical properties, such as high molecular weight, short half-life, and the requirement of a sustained plasma level for the desired therapeutic effect, which is liable to physical and chemical instability by gastric enzymes and harsh acidic environment, as well as first pass metabolism. In order to maintain the ACE inhibitory activity, it is necessary to release a minimum quantity of hydrolysate during gastric digestion and release the maximum amount during intestinal absorption. Treatment 1 released 60.1% of protein during intestinal conditions and protein maintained an IC_50_ of 2.9 mg/mL. Beads obtained with the blend of flamboyant carboxymethylated gum/sodium alginate could be used to control the delivery of hydrolysates with potential use as therapeutic and preventive agents. 

## 4. Conclusions

A blend of *Delonix regia* carboxymethylated gum/sodium alginate is effective in encapsulating *Phaseolus lunatus* hydrolysate with ACE inhibitory activity, suggesting that it could be an effective delivery system in the preventative treatment of hypertension. Although further research is needed regarding the optimization of encapsulation, *in vivo* studies of delivery and the hypotensive effect of this non-conventional gum blend show that it provides an effective encapsulation system for hydrolysates with biological activity, which could be used as a nutraceutical or therapeutic agent. 

## Figures and Tables

**Table 1:** 2^2^ factorial design used for *Phaseolus lunatus* hydrolysate encapsulation with flamboyant carboxymethylated gum/alginate and protein encapsulation efficiency.

Treatment	CaCl_2_ (mM)	pH	Encapsulation efficiency (%)
1	1.0	4	71.7^c^
2	3.0	4	67.3^b^
3	1.0	10	78.4^d^
4	3.0	10	71.8^c^
5	2.0	7	53.1^a^
6	2.0	7	53.3^a^
7	2.0	7	51.8^a^
8	2.0	7	52.0^a^

Different superscript letters indicate statistical difference (*P* < 0.05).

**Table 2:** Protein released and IC_50_ values at gastric and intestinal simulation conditions.

Treatment	Protein released (%)		IC_50_ (mg/mL)	
	Gastric pH 1.2, 37°C	Intestinal pH 6.8, 37°C	GpH	IpH
1	16.4^c^	60.1^c^	3.8^a^	2.9^c^
2	10.5^a^	39.4^b^	4.1^b^	3.3^b^
3	73.1^d^	20.2^a^	3.1^c^	3.8^a^
4	14.2^b^	40.3^b^	3.9^a^	3.8^a^
CP	17.9^c^	40.7^b^	3.8^a^	3.7^a^

Different superscript letters in the same column indicate statistical difference (*P* < 0.05).
